# Characterising experiences with acute myeloid leukaemia using an Instagram content analysis

**DOI:** 10.1371/journal.pone.0250641

**Published:** 2021-05-03

**Authors:** Catriona Parker, Ella Zomer, Danny Liew, Darshini Ayton

**Affiliations:** 1 School of Public Health and Preventive Medicine, Monash University, Melbourne, Victoria, Australia; 2 Department of Haematology, The Alfred Hospital, Melbourne, Victoria, Australia; Brown University, UNITED STATES

## Abstract

Instagram has more than one billion monthly users, which presents a unique research opportunity particularly in rare diseases or hard to reach populations. This study focuses on acute myeloid leukaemia, a rare haematological malignancy and aims to characterise who posts acute myeloid leukaemia-related content and the type of content created. The findings can provide information and a method for future studies, particularly those focused on online or social media based interventions. Acute myeloid leukaemia-related Instagram posts were identified by searching specific and relevant hashtags (#). A content analysis systematically classified themes in the data. A convenience sample of 100 posts (138 photos) were manually extracted and coded. Data are described using descriptive statistics and demonstrated by qualitative examples. The most frequent users in our sample were patients (66%), patient support networks (24%) and professional organisations (10%). Patients who were communicating their health update (31%) were the most frequently posted content and 25% of these posts described a symptom experience. Our findings demonstrate that patients and their support networks are frequenting Instagram and therefore may be able to receive and benefit from tailored intervention, however there is an identified gap in health-organisations participating in this virtual online community.

## Introduction

Foreseeably, past generations of patients have used their physicians as the key source of health related information, however, there is evidence that people are increasingly turning to the Internet to supplement their information needs [1]. For example, a Swedish study found that just over three-quarters (76.2%) of people diagnosed with cancer accessed the Internet for cancer-related information and more than one quarter used social media relating to their health [2]. Patients commonly report using webpages, blogs, interactive forums and social media to obtain information to help make informed decisions, find practical information or answers to health related questions, stay in touch with others, and share experiences [2, 3].

Social media has become ubiquitous to our lives where we share, connect and communicate our experiences with friends, family, organisations and people otherwise unknown to us. Worldwide, approximately 2.5 billion people use social media and almost two-thirds of American adults use social networking sites: an almost ten-fold increase over the past decade [4]. The portability of these websites via mobile applications has no-doubt accelerated their uptake and allows for the capture of life’s most ephemeral events. The differences in user demographics that are seen between platforms (such as age, ethnicity, gender, education or income), lend themselves to being targeted for various health campaigns, health promotions or health research seeking to reach different audiences [5, 6]. Social media data collection foreseeably provides large-scale and easily accessible data for patient reported information, particularly when compared with traditional patient-focused data collection methods [7].

One of the most popular social media platforms, Instagram, has almost one billion active monthly users [8]. Instagram is differentiated from other social media platforms by user-posts’ being dominated by a photo. Most users choose to add accompanying text to their photos as well as tags or labels, termed ‘hashtags’ denoted as #*label*, (e.g. #cancer). The accompanying hashtags provide a method of grouping photos to create virtual social communities of similarly themed content or purpose and allows users to easily connect and share content.

Acute myeloid leukaemia (AML) is a relatively rare and aggressive blood cancer that can occur at any age [9, 10]. The standard treatment is immediate intensive chemotherapy, requiring lengthy hospital stays [11]. Additionally, research shows most patients have a reduced quality of life and persistent side effects or symptoms even after the completion of therapy or in remission [12–14].

AML makes up less than 1% of all cancer diagnoses per year, making research challenging to accrue participants, particularly in young adulthood where incidence is at its lowest [9, 10]. However due to the popularity of Instagram, particularly in early adulthood [15] and the search functionality of hashtags, the Instagram platform presents an opportunity for proposing unique research questions, particularly those focused on rare-diseases (as with AML), or research with participants that are traditionally difficult to access. Despite the popularity of Instagram, and the unique participant group it can reach, little health research has been undertaking using this platform [1].

Given large numbers of people with cancer are accessing online health-related messages and the relative absence of Instagram research, this exploratory study will be the first to characterise AML-related content on Instagram; specifically who is posting AML-related content and what types of content are being posted. Characterising AML-related content on social media could be useful for targeting people most likely to benefit from health messages, interventions, or support. Using Instagram for this type of extant research has the potential to provide unique insights into the lived experience, as well as observing individuals providing or receiving support through virtual communities and the sharing of health-related information. Additionally we detail a method potentially of interest to other researchers.

## Materials and methods

The methods outlined in this paper adhered to Instagram’s terms of service at the time of the research and all the content analysed in this study was publicly available on Instagram (available at www.instagram.com/instagram). However the data is not owned by the authors and they do not have permission for reproduction of the data used in the analysis. Ethical approval for the study was granted from Monash University, Human and Research Ethics Committee (Project ID 18540) and included a waiver of consent (no individual consent was necessary from Instagram users). The ethics approval prohibits the publication of data that may inadvertently identify any individual.

### Data collection

Instagram is primarily a mobile application but has a desktop website with limited functionality. Only the website accessible version of Instagram was used in this study, to ensure complete separation between the researchers and their private accounts. This also ensured that only publicly available posts were being accessed (no Instagram account or login required). One hundred posts were chosen as a convenient number for a time-consuming manual exploratory method. The posts were found by using Instagram’s search bar at the top of the webpage using hashtags that had been previously scoped as being used by people with AML: #acutemyeloidleukemia, #acutemyeloidleukaemia and #amlsurvivor. Each hashtag was searched for separately.

Posts were excluded from the study if they were videos (we were unable to extract these using our context extraction method), had non-English accompanying text or the subject matter was focused on children’s cancer. Children’s cancer was able to be determined by accompanying hashtag, such as #childhoodcancersucks or though examining the post photo and/or the accompanying text.

This study was undertaken after Instagram removed the automatic application program interface (API), which allowed for automation in downloads and much of the accompanying meta-data. Therefore, we detail a manual method of data extraction that may be of use to other researchers. This manual method allowed for retrospective capture for all eligible posts made over seven consecutive days in February 2019; eight consecutive days in April 2019 and twelve consecutive days in May 2019, to obtain a consecutive sample of 100 posts during the collection periods. Posts in chronological order (as opposed to most popular) can be found by scrolling past the initial “top posts” to the “most recent”. It is was these most recent posts that were accessed taking note of the date of the post to ensure it complied with our sampling time-frame. This sampling method was used to avoid awareness campaigns or trending content, (which may generate atypical Instagram posts and traffic), cultural and ethnic influences between users’ geographical location and for researcher convenience. One hundred posts was deemed to be sufficient given practicality of methods employed and the rarity of AML for an initial exploratory study.

As a user can modify or delete the content or their Instagram account, a screenshot was taken of the post and the user profile, thereby creating a ‘post-record’, which became the main unit of analysis. The post-record was made using Microsoft Word. We analysed the content of a post to include both the photo and the accompanying text and hashtags but excluded subsequent comments (and hashtags) that were made by either the ‘post-owner’ or other users.

For each post, basic data points were gathered about the user and the post: age and gender of the user (self-reported in the user profile), and country of origin data by using the location specified as part of the post or contained in the user profile, as well as post-specific information (description of the photo/s, accompanying text and hashtags and the number of likes and comments etc.). We also captured the username, but as duplicates became apparent, we adopted our identification system for each post to be able to distinguish between different posts from the same users.

Whilst the Instagram posts are publically available the data cannot be reproduced to comply with the Instagram terms of service, comply with the ethical approval of this study and to protect the privacy of the individuals posting on Instagram.

### Data analysis

**Fig 1:**
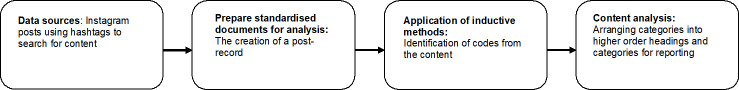
Method process, adapted from a mixed methods social network analysis framework [16].

We used an adapted mixed-method social network model to frame our analysis [16]. The model describes sourcing data (Instagram), constructing the data (organising and preparing for analysis) and analysing the data (using network analysis or linear modelling). The framework was appropriate as the study was exploratory and observational and employed a content analysis, however it was modified as we did not employ the network analysis or linear modelling. Fig 1 demonstrates our approach.

The content analysis is ideal for exploratory research, as this method seeks to unobtrusively explore the explicit description of the communication and the trends, patterns and frequency of this communication found within data [17, 18]. No *a priori* coding was developed owing to an absence of literature relating to the content of AML on Instagram. An inductive approach was employed to identify frequently occurring content categories and themes [18].

**Fig 2:**
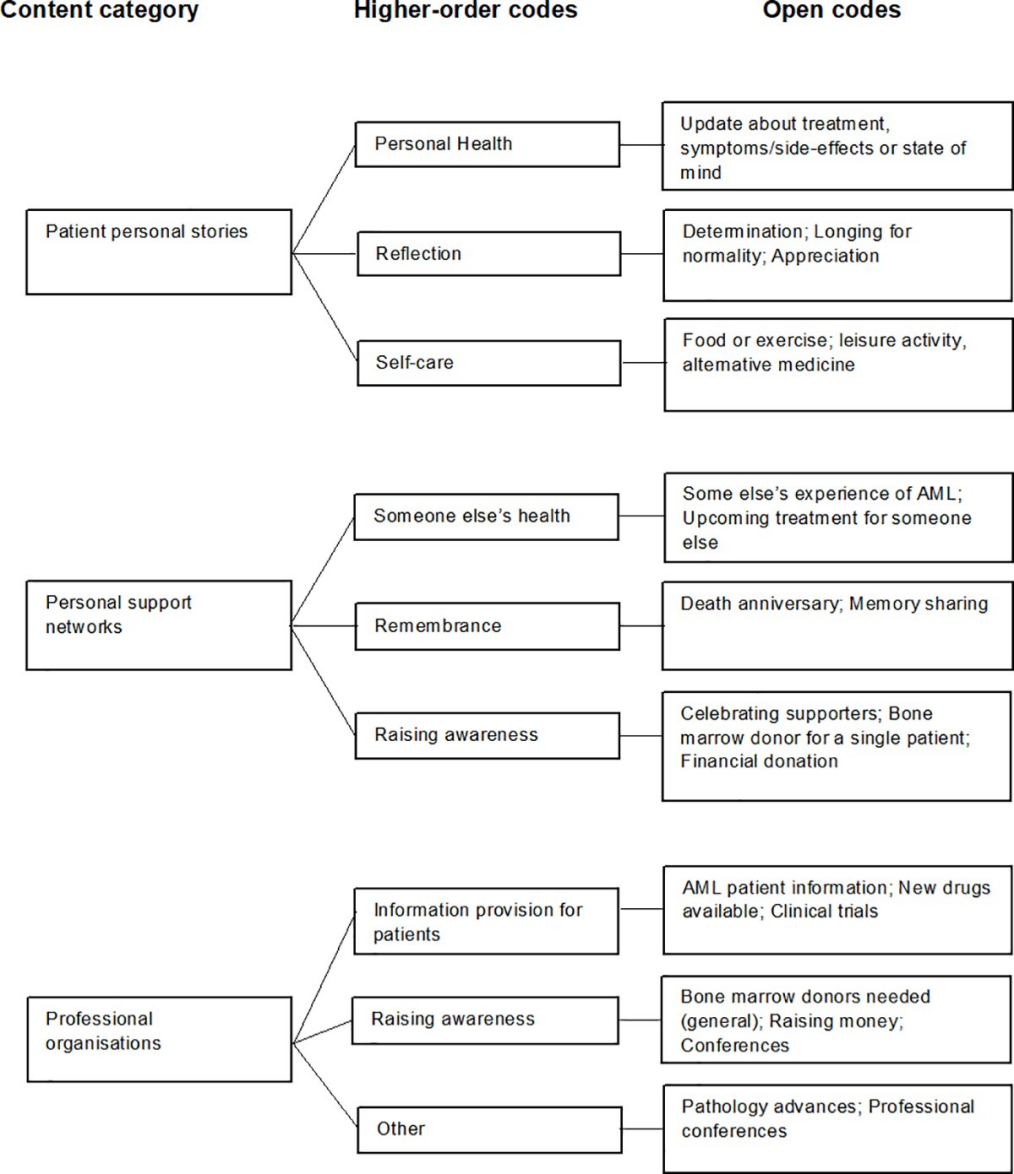
Process of generating categories.

In brief the process included open coding, creating higher headings and then categories. After reviewing the post records multiple times, open codes were developed in a consultative and iterative process of reviewing the first ten post-records, at which time an open coding scheme was generated, that we thought could be applied to the whole data set [19]. A further ten posts were classified according to our coding scheme and codes were refined as necessary. The first initial ten posts were re-coded as per this scheme. This process was repeated twice, until we had a open coding scheme (after coding 40 posts) that could be applied to the entire data set. Higher order headings were then able to be developed from the open codes using researcher interpretation as to which codes belong in each higher order heading and then into categories (Fig 2) [20]. The process was undertaken by two reviewers and any discordance in coding between reviewers was discussed to reach consensus [18].

After the process was finished and the researchers were reflecting on the findings, we went back and coded for a single theme: ‘hope and/or gratitude’, as the researchers felt that even though this was outside the content analysis it was an interesting finding relevant to the research. This theme was based on the researchers’ interpretation of the image and accompanying text.

Most data were expressed as both means with standard deviations, medians with interquartile ranges (IQRs), as well as frequency and range because the content and distribution varied considerably. Microsoft Excel was used for these descriptive statistics.

## Results

During the search period, almost all posts were found using #acutemyeloidleukemia (94%). During the window of analysis, 51 unique users posted content and 16 of these posted more than once resulting in the analysis of 100 posts, consisting of 138 photos—one post can contain up to 10 photos.

Age and gender were mostly unavailable. Only two profiles stated age but we have chosen to conceal this for re-identification protection. Gender was rarely specified in the user profile, and we deemed it unreliable to discern gender either, from self-description (e.g. mom, wife etc.), appearance or socially gender-normative names, so this has not been reported. As shown in Table 1, we were mostly unable to determine the country of the post origin for most users (34/51).

**Table 1:** Country of post origin of the post or user account (n = 51).

Country	Frequency n (%)
**United States**	11 (22)
**Canada**	1 (2)
**United Kingdom**	3 (6)
**Hong Kong**	1 (2)
**Malaysia**	1 (2)
**Unknown**	34 (66)

We identified three user categories from the data: patient personal stories, personal support networks and professional organisations (Table 2). The most frequent users were patients themselves (66% of the posts), followed by personal support networks that we interpreted as family and friends (24% of the posts) and lastly professional organisations (10% of the posts).

**Table 2:** Frequency of posts and photos in each user category.

User categories	Number of posts (n = 100) n (%)	Number of photos (n = 138) n (%)
Patient personal stories	66 (66)	99 (72)
Personal support networks	24 (24)	26 (19)
Professional organisations	10 (10)	13 (9)

As shown in Table 3, the most frequent content posted in the analysis was patients communicating their health update (31% of the whole sample). The majority of posts made by personal support networks was a health update on behalf of a patient (50% of the personal support networks user category). Professional organisations only accounted for 10% of the total sample and the majority of the content was either patient information provision (40% of the posts) or raising disease awareness (50% of the posts).

**Table 3:** The content classification frequency by user category and content classification.

User category	Content classification	Frequency of posts for each user category n (%)	Frequency of content classification for whole sample (n = 100) %
Patient personal stories (n = 66)	Personal health	31 (47)	31
	Reflection	24 (36)	24
	Self-care	11 (17)	11
Personal support networks (n = 24)	Someone else’s health	12 (50)	12
	Remembrance	4 (17)	4
	Raising awareness	8 (33)	8
Professional organisations (n = 10)	Information provision for patients	4 (40)	4
	Raising awareness	5 (50)	5
	Other	1 (10)	1

The 10 organisational posts comprised of seven users and thirteen photos. Five of the seven users had an unknown country of origin, while one was based in the United States and the other in the United Kingdom as discerned from their profile or dot-org websites.

One-quarter of all posts detailed symptoms that were being experienced by patients and 19/25 posts containing symptoms came from patients with the remaining posts being made by personal support networks. Please note to the protect privacy of individuals (for example via reverse identification), the quotes chosen below have been altered to encompass the overall sentiment of the quote [21].

“#selfie #nofilter long term chemotherapy effects have mostly subsided. Still can’t shake that #red eye…” (picture of a person smiling into the camera). ***Patient personal story***.

“…Hubby had platelets to fix his bleeding gums…” (picture of a person sitting upright in bed, surrounded by medical equipment) ***Personal support networks***.

Likes and comments were used as a proxy measure for engagement (Table 4). Overall there was between three and 394 likes and between zero and 54 comments. There was little engagement with organisational posts as measured by ‘likes’ and comments. There were between eleven and 41 likes on the posts and five posts had no comments.

**Table 4:** Engagement with posts by user category as measured by likes and comments.

User category	Likes			Comments		
	**Mean (SD)**	**Median (IQR)**	**Range**	**Mean (SD)**	**Median (IQR)**	**Range**
**Patient personal stories**	67.41 (68.61)	36.5 (51.5)	251	7.47 (10.16)	4 (8.75)	54
**Personal support networks**	79.71 (41)	91.87 (52.75)	391	5.58 (7.9)	3 (4.75)	31
**Professional organisations**	28.9 (8.64)	31 (12.75)	30	1 (1)	1 (1)	11
**All posts**	66.51 (73.59)	35 (50)	391	6.43 (9.35)	3(6)	54

Additionally, throughout the analysis, we noticed a prominent theme of hope often accompanied by gratitude, in the posts, either implicitly but commonly through the use of the accompanying text or hashtags (e.g. #gratitude or #grateful or #thankyou or #hopeful). Almost half (49%) of all the posts demonstrated this theme hope and/or gratitude. Thirty-four of these were made by the user category of patient personal stories, eleven by personal support networks and four by professional organisations.

“…Each day has something good in it, even on the toughest of days…” (Image of a motivational meme) ***Patient personal story***.

## Discussion

While much of the social media cancer communication research has focused on Facebook and Twitter, very few studies have focused on Instagram, particularly with a focus on such an emotionally and physically burdensome cancer like AML. Instagram differs from Facebook and Twitter by incorporating visual cancer communication and to our knowledge this is the first study to describe the content of Instagram communication concerning AML, thereby addressing this research gap. The novel method we have outlined is most useful for other investigators looking to utilise social media in the their research and our findings should be considered in the context of the limitations of our methods.

Our exploratory descriptive research showed in our sample, that people with AML communicating personal health updates, was the most common content being posted about AML on Instagram. Personal story sharing related to AML was also prominent by the personal support networks user category of people with AML. This finding was congruent with other Instagram disease-related research [3, 22, 23].

Why people tell such personal stories through Instagram may be explained by social media use being linked with patient empowerment through improved self-management and enhanced psychological and subjective well-being [1, 3]. These benefits may be obtained through real or perceived social connectedness of users of Instagram where they feel a sense of intimacy through sharing or social support, through community [24–26]. By posting intimate stories, users may also provide and receive social and emotional support through these virtual online communities [23]. This is further supported by the high prevalence of hope and/or gratitude in our data, where Steffen et al found in a study of advanced lung cancer patients, that hope may be important in providing support to social and role functioning, irrespective of physical symptom severity [27]. In sentiment analysis, Cho and colleagues also found hope was the most commonly expressed emotion in their melanoma study [23]. Whether hope is a common finding on social media contained to people with a malignant disease remains unknown.

In contrast to a Facebook content analysis including breast, prostate and other reproductive cancers, Instagram users concerned with AML do not appear to be information seeking, which may be due to the inherent functionalities of the platform [28]. This means that health professionals, researchers and professional organisations should endeavour to tailor their communication respectively to the most appropriate platform. However, if users are predominately seeking or providing support through personal storytelling, Instagram presents an opportunity for health providers and other organisations tasked with awareness-raising or support and wellbeing. Furthermore, it is likely patients and their friends and family are highly motivated to sustain the engagement with cancer communication initiated by reputable professional organisations [28, 29]. It is worth noting, we were unable to identify any health providers (individually or part of a health facility) posting during our data extraction period. The content of what patients communicate via social media outside the immediate doctor-patient consult provides an unique viewpoint unhindered by bustling waiting rooms or the interpretation of clinicians, to contextualise patient experience and decision making [7]. In our study, only about 10% of posts were organisational suggesting that Instagram may represent an untapped resource for cancer support communities and awareness campaigns. This suggestion possibly holds relevance for all cancer types. Furthermore, public awareness is particularly relevant for malignant haematological diseases where up to 70% of patients need to seek bone marrow transplant donors outside of their family and only 7% of the American population are registered bone marrow donors [30]. Increasing public awareness through emotional appeal and capitalising on hope as a concept, may increase the number of registered donors to ensure sufficient diversity in the donor pool to meet the patient demand for bone marrow transplant [31, 32].

Social media research can complement other research methods: Crawford et al used YouTube to complement a literature review about the patient experience of haematological malignancies and found that YouTube provided supplementary information that highlighted the multifactorial experiences of patients that may not have been otherwise apparent through traditional research methods [7].

Certainly some individual healthcare professionals can and are using social media. A recent Italian study of neurologists showed that 56% of the sample used social media to have direct contact with patients and most of these health professionals were in favour of this communication method [33]. Instagram may provide an opportunity for clinican-led content that is trustworthy and appeals to patients, yet clinician-led social media posts are lacking, yet [34]. Moorhead et al. [35] suggests that both health professionals and their patients may need training to maximise the use of social media in their healthcare interaction. However, as yet it remains unknown how effective social media can be in its’ perceived role in healthcare [35] and how this applies to inherently passive platforms such as Instagram where interactivity between user and viewer is limited.

Given the popularity of Instagram and the potential reach of posts, further research is warranted to understand the implications of online visual communication and how this information can be harnessed to improve health communication, patient experience and the experience of healthcare and balancing this with minimising the perpetuation of misinformation to vulnerable individuals.

Our study is not without limitations: critics rightfully observe that Instagram is often curated and may not reflect real life—experiences are complex and Instagram is a snapshot in time. Additionally, our sample may not reflect the breadth of posts due to our sample size, which was limited by the practicality of employing a manual method and resourcing. The manual method we employed and limitations in the search function also meant the study was unable to capture videos and Instagram stories (which are only available for 24 hours from posting). The sample used in this study had many users posting multiple times, potentially meaning our results may be less diverse and biased towards fewer individual experiences of AML. The retrospective nature of this study only allowed for the capture of data about age, gender and location that the user chose to share and it is therefore unknown whether there are dominating age groups, gender or country of origin in our analysis.

The strengths of this study are that we have demonstrated a unique and innovative way to potentially reach and/or observe hard to reach populations or people suffering rare conditions. Additionally, photos are a unique and expressive medium not conventionally used in cancer support services so other researchers with appropriate research question could also choose to employ an interactive image-based study design.

## Conclusion

This exploratory study, presents a novel method whereby we have characterised AML-related Instagram content that contributes to the understanding of how social media fits into the lives of people affected by AML. Our results suggest that social media may have a role to play particularly for social connectedness and support and that there is a potential role to play for health professionals and health organisations.

Further research should focus on exploring the feasibility and effectiveness of targeted awareness campaigns, as well as deploying support networks or health interventions to aid people by providing or seeking support.
